# Enhanced photocatalytic degradation of Congo red by a BiVO_4_/ZnIn_2_S_4_ composite: performance and mechanism

**DOI:** 10.1039/d6ra00765a

**Published:** 2026-04-27

**Authors:** Xinyue Liu, Yixin Zhang, Jiaxing Yu, Ming Li

**Affiliations:** a Nanjing-Helsinki Institute in Atmospheric and Earth System Sciences, Nanjing University Suzhou 215163 China; b College of Forestry, Northeast Forestry University Harbin 150040 China liming1986@nefu.edu.cn +86-451-82192120

## Abstract

High-performance visible-light-driven photocatalysts have emerged as a research hotspot in environmental pollution; however, the photocatalytic degradation mechanisms of Congo red using bismuth semiconductor composites are not fully understood. Herein, BiVO_4_/ZnIn_2_S_4_ was successfully synthesized *via* a hydrothermal method, and the photocatalytic performance of the composite was assessed by carrying out photocatalysis experiments to decompose Congo red. The phase composition, carrier separation capability, interface electron interactions, and morphological features were characterized by XRD, PL, XPS, and TEM analyses, respectively. The results showed that a Z-scheme heterostructure was successfully constructed between the BiVO_4_ and ZnIn_2_S_4_. Electrons in the conduction band of BiVO_4_ migrated to the valence band of ZnIn_2_S_4_, which effectively enhanced the separation of photogenerated charge carriers and improved the degradation capability. Compared with pure BiVO_4_, 7% BiVO_4_/ZnIn_2_S_4_ displayed superior photocatalytic capability and could completely remove Congo red (100 mg L^−1^) in 60 min under visible light. Radical trapping experiments combined with electron paramagnetic resonance characterization revealed that the superoxide anion (˙O_2_^−^) and holes (h^+^) acted as the key reactive species driving Congo red degradation. In addition, six cycles of experiments were performed to verify that the BiVO_4_/ZnIn_2_S_4_ composite retains its high stability. This study provides a feasible strategy for fabricating effective photocatalysts to treat organic pollutants in wastewater.

## Abstract 2

BiVO_4_/ZnIn_2_S_4_ was synthesized *via* a hydrothermal method, which enabled rapid decomposing Congo red under visible light, providing a feasible strategy for fabricating high-efficiency photocatalysts in water treatment.

## Graphical abstract

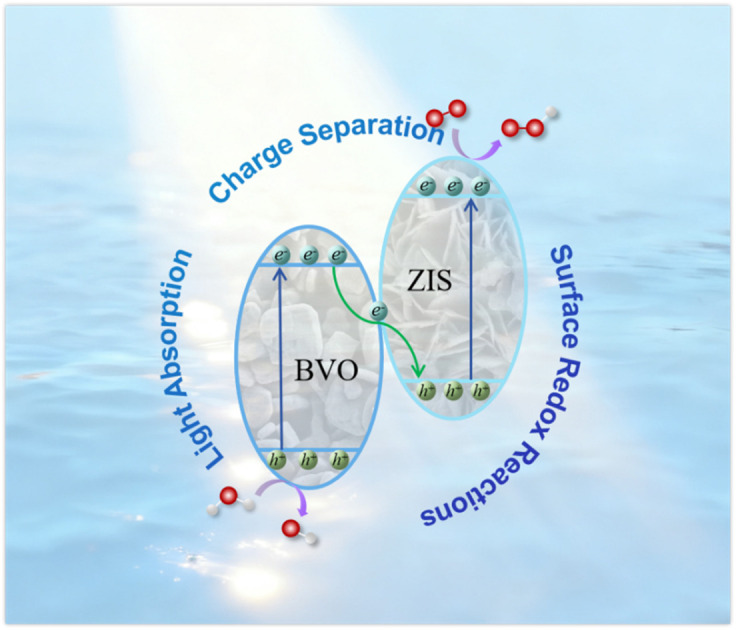

## 1 Introduction

Water is essential for life on Earth and the basic guarantee of human reproduction and life. Unfortunately, due to the rapid development of printing and dyeing industries, the discharge of dyestuff wastewater has dramatically increased.^1^ Wastewater containing synthetic organic dyes can harmfully affect the structure of aquatic communities.^2^ As a typical benzidine direct azo dye, Congo red is widely used in the textile, paper-making, rubber, and plastics industries; however, it can induce allergic reactions and is metabolically converted into benzidine. Due to the high stability of Congo red,^3^ removing it from industrial wastewater is extremely difficult. To ensure the safety of drinking water, wastewater containing toxic substances should be treated before being released into the environment. Various biological and physicochemical methods, *e.g.*, membrane filtration,^4^ precipitates,^5^ ion exchange,^6^ biodegradation,^7^ and adsorption^8^ have been employed to treat wastewater. However, these conventional approaches are inefficient and have limitations; *e.g.*, membrane filtration and precipitate techniques are expensive and produce hazardous secondary contaminants and waste materials.^9^ Although anaerobic digestion is cost-effective, it struggles to fully mineralize the recalcitrant organic compounds such as Congo red due to slow microbial metabolic rates and poor tolerance to toxic pollutants, and it is also highly susceptible to fluctuations in environmental conditions.^10^ Advanced oxidation processes (such as the Fenton process^11^ and photocatalysis) have been demonstrated to be the most effective to fully eliminate organic pollutants from wastewater. The Fenton process has been widely applied in this field due to its strong oxidizing capacity.^12^ Furthermore, in recent years, photocatalytic technology has been widely used to remove pollutants, produce hydrogen and ammonia, and regulate the nitrogen cycle^13^ due to its excellent oxidation capacity, environmental friendliness, compatibility, long-term high efficiency, and economic sustainability.^14^ The photocatalytic process is independent of microbial activity and can rapidly mineralize organic pollutants under visible light, showing a broader degradation spectrum, faster reaction rates, and stronger resistance to interference. Therefore, the development of novel photocatalysts can provide an effective technique to deal with wastewater pollution. Among the visible-light photocatalytic materials, bismuth-based photocatalysts are considered outstanding visible-light-driven photocatalytic materials due to their narrow band gap (*E*_g_) of less than 3.0 eV.^15^ Notably, bismuth vanadate (BiVO_4_) has attracted great research attention because of the unique optical, electrical properties, and promising photocatalytic performance. BiVO_4_ effectively absorbs visible light, significantly improving its solar energy utilization efficiency and overcoming the low efficiency limitations of UV photocatalysts such as TiO_2_. However, its catalytic performance is constrained by a narrow specific surface area and suboptimal separation of photogenerated electron–hole pairs. To achieve the separation of photogenerated carriers, the construction of heterojunctions has become a novel research hotspot. BiVO_4_ possesses sufficient valence-band potential to generate holes and reactive oxygen species with robust oxidative capacity.^16^ Accordingly, a range of BiVO_4_-based catalysts have been developed for diverse applications; *e.g.*, Fe_3_N/BiVO_4_ was used for H_2_ generation,^17^ BiVO_4_/g-C_3_N_4_/rGO and BiVO_4_/TiS_2_ were employed to enhance pollution degradation,^18^ CdS/BiVO_4_ was adopted for CO_2_ reduction,^19^ and MoS_2_/BiVO_4_ was applied for bacterial disinfection.^20^

Zinc indium sulfide (ZnIn_2_S_4_) is a metal–thiolate material with a layered spinel structure that has drawn considerable interest due to its prominent photoelectric response and environmental stability.^21^ The interlayer bonding *via* van der Waals forces provides a rapid transport channel for photogenerated carriers, effectively suppressing bulk recombination. Z-scheme Bi_2_S_3_/ZnIn_2_S_4_ heterostructures have been successfully synthesized to enhance charge separation and transport rate, boosting photogenerated carrier separation and improving methylene blue photodegradation efficiency.^22^ Notably, the BiVO_4_/ZnIn_2_S_4_ Z-scheme heterojunction photocatalytic system can be further activated by introducing NaBH_4_ and peroxymonosulfate. Analysis of the band structure and charge transfer mechanism indicates that the composite system has excellent potential for photocatalytic coupling with advanced oxidation processes ^23,24^. The *E*_g_ of ZnIn_2_S_4_ is 2.6 eV, and the conduction band (CB) value is below zero, which means that it can absorb more visible light.^25^ Given that BiVO_4_ and ZnIn_2_S_4_ have similar *E*_g_ values, and the CB value of ZnIn_2_S_4_ is much lower than that of BiVO_4_, these two materials possess a matched band structure and complementary light absorption ranges that are ideal for constructing suitable heterojunctions to further improve the photocatalytic performance of BiVO_4_.

Herein, a direct Z-scheme heterostructure BiVO_4_/ZnIn_2_S_4_ photocatalyst was fabricated. Subsequently, photocatalytic degradation of Congo red by the BiVO_4_/ZnIn_2_S_4_ photocatalyst was investigated. A possible photocatalytic mechanism describing the BiVO_4_/ZnIn_2_S_4_ system was also proposed.

## 2 Experiment

### 2.1 Synthesis of catalysts

Bi(NO_3_)_3_·5H_2_O (4.85 g, 0.001 mol) was dissolved in 20 mL of HNO_3_ solution, which was recorded as solution A. Furthermore, NH_4_VO_3_ (1.17 g, 0.001 mol) and EDTA (1.99 g, 0.0068 mol) were dissolved in NaOH solution (40 mol L^−1^, 20 mL) and labeled as solution B. Subsequently, solution B was gradually added to solution A, the mixture was stirred for 30 minutes, and its pH was adjusted to 5. Then the mixture (70 mL) was slowly injected into a 100 mL Teflon-lined stainless-steel autoclave and hydrothermally treated at 180 °C for 24 hours. After heating, the sediments were collected *via* filtration, rinsed with water and ethanol to eliminate impurities, and finally dried at 65 °C to yield pure BiVO_4_.^26^

ZnCl_2_ (0.136 g, 1 mmol), InCl_3_·4H_2_O (0.586 g, 2 mmol), and CH_3_CSNH_2_ (TAA) (0.3 g, 4 mmol) were fully dissolved in deionized water. A defined amount of the pre-synthesized BiVO_4_ solid was dispersed into the above-mentioned mixture to attain BiVO_4_/ZnIn_2_S_4_ samples with weight ratios of 7%, 15%, and 25%. After sonicating for 1 hour, the mixture (40 mL) was slowly injected into a Teflon-lined stainless-steel autoclave and hydrothermally treated at 180 °C for 12 hours. When the hydrothermal reaction was complete, the resulting precipitates were collected by filtration, rinsed with deionized water and ethanol to eliminate impurities, and finally dried at 65 °C to obtain a BiVO_4_/ZnIn_2_S_4_^27,28^.

### 2.2 Photocatalytic experiments

Catalysts (50 mg) with different ratios were dispensed into separate solutions of Congo red (100 mL and 400 mg L^−1^). Before light irradiation, the suspensions were stirred in the dark for 30 minutes to establish adsorption–desorption equilibrium between the catalyst and Congo red. Subsequently, degradation experiments were conducted using a cylindrical photochemical reactor fitted with a simulated sunlight source. The Congo red solution was decomposed under irradiation from a 300 W xenon lamp (equipped with a 420 nm cutoff filter). Every 10 minutes, 2 mL of liquid was taken and filtered through a 0.22 µm membrane. The concentration of Congo red was measured using a UV-Vis spectrophotometer (Shimadzu UV3600, Japan).

To further clarify the major reactive species dominating the photocatalytic reaction, three scavengers were introduced: benzoquinone (BQ, 0.1 mmol L^−1^), triethanolamine (TEOA, 10 mmol L^−1^), and isopropanol (IPA, 10 mmol L^−1^) to target superoxide radicals (˙O_2_^−^), holes (h^+^), and hydroxyl radicals (˙OH), respectively.

### 2.3 Characterization

The crystalline phase of the samples was studied by X-ray diffraction (XRD, TD-3500, China) . To study the surface morphology and microstructure, scanning electron microscopy (SEM, Gemini 300, ZEISS, Germany), high-resolution transmission electron microscopy (TEM, JEOL JEM-2100F, Japan), and energy-dispersive spectrometry (EDS) analyses were utilized. Additionally, X-ray photoelectron spectroscopy (XPS, Thermo Fisher, USA) was adopted to verify the samples' elemental compositions and surface. A Brunauer–Emmett–Teller (BET, Quantachrome, Autosorb iQ) instrument was used to determine the pore properties and specific surface areas. Functional group information was characterized *via* Fourier-transform infrared spectroscopy (FT-IR, Nicolet Nexus 670, USA) analysis. Photoluminescence (PL) spectra were recorded with a Hitachi fluorescence spectrophotometer (FLS980, UK). Trapping experiments and electron paramagnetic resonance (EPR, Shimadzu, Japan) measurements were conducted to identify the active species participating in the photocatalytic reaction. Finally, the UV-vis diffuse reflectance spectra (DRS) of the synthesized photocatalysts were collected with a UV-vis spectrophotometer (Shimadzu UV3600, Japan).

## 3 Results and discussion

### 3.1 Characterization of catalysts

**Fig. 1:**
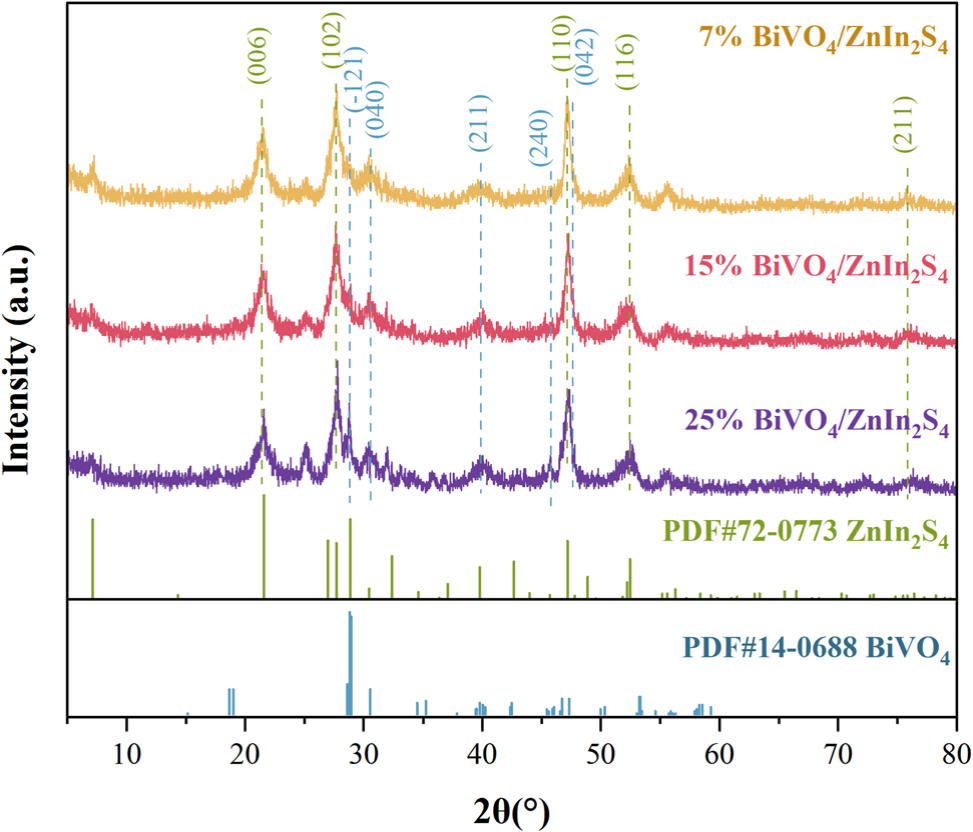
XRD patterns of BiVO_4_/Znln_2_S_4_.

Fig. 1 presents the XRD spectra of BiVO_4_/ZnIn_2_S_4_ composite samples with different BiVO_4_ mass fractions. The patterns of the composite materials exhibit the characteristic diffraction peaks corresponding to the monoclinic phase BiVO_4_ (ref. 29) (PDF# 14-0688) and the hexagonal crystal type ZnIn_2_S_4_ (ref. 30) (PDF# 72-0773). The distinct peaks observed at 2*θ* values of 28.82°, 30.54°, 39.78°, 46.71°, and 47.30° matched with the (−121), (040), (211), (240), and (042) planes of BiVO_4_, respectively; meanwhile, the distinct peaks at 21.58°, 27.69°, 47.17°, 52.21°, 52.44°, and 75.47° corresponded to the (006), (102), (110), (112), (116), and (211) crystal planes of ZnIn_2_S_4_, respectively, confirming that the composite contained BiVO_4_/ZnIn_2_S_4_.

**Fig. 2:**
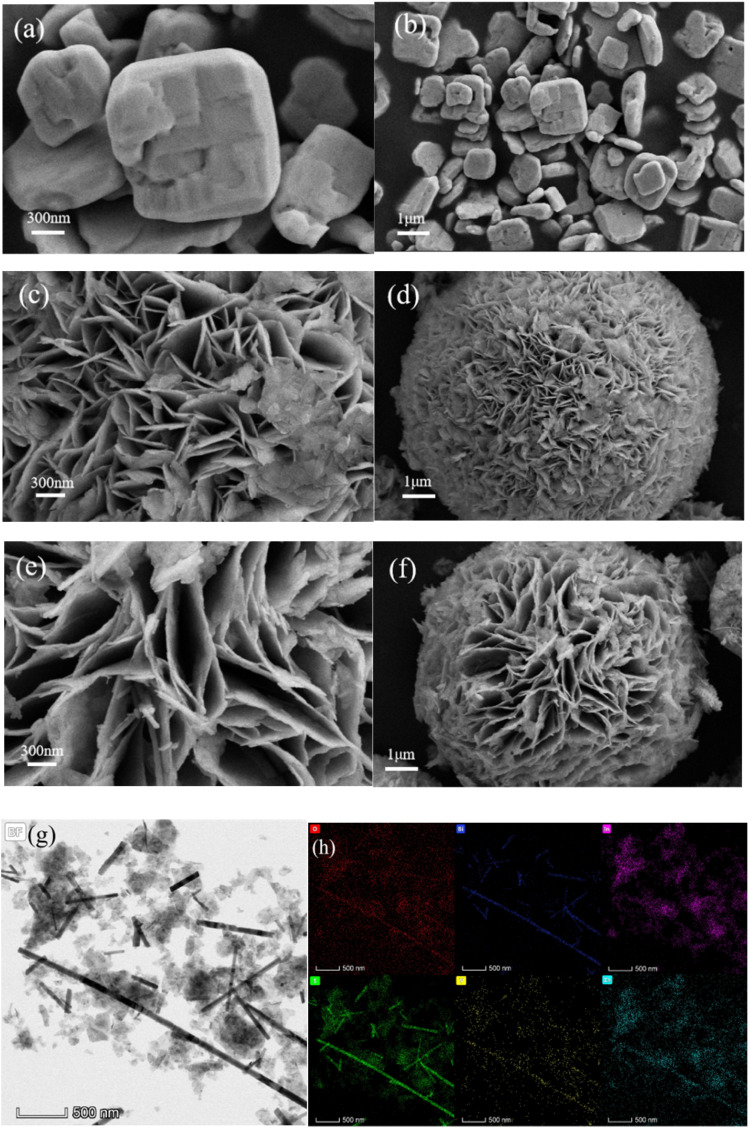
SEM images of the photocatalysts: BiVO_4_ (a and b), ZnIn_2_S_4_ (c and d), and BiVO_4_/ZnIn_2_S_4_ (e and f). TEM bright-field image (g) and the corresponding EDS elemental mapping (h) of BiVO_4_/ZnIn_2_S_4_.

As shown in Fig. 2a, decahedral BiVO_4_ presents a smooth monoclinic phase. As shown in Fig. 2b, ZnIn_2_S_4_ presents a folded layer, which would contribute to an expanded specific surface area and an increased number of active sites, enhancing the separation efficiency between electrons and holes. As can be seen in Fig. 2c, BiVO_4_ is anchored to the ZnIn_2_S_4_ surface, forming tight contacts without changing the morphology of the ZnIn_2_S_4_. However, the BiVO_4_/ZnIn_2_S_4_ composite has a coarser surface than pure ZnIn_2_S_4_, which expands the reaction area and enhances its photocatalytic activity. As shown in Fig. 2g, the sample exhibits distinct rod-like structures with uniform distribution. These structures have been confirmed as BiVO_4_ and are well-dispersed on the surface of the ZnIn_2_S_4_ matrix. The rods exhibit a typical length of several hundred nanometers and a diameter of tens of nanometers. This unique rod-like morphology not only increases the specific surface area of the composite, providing more active sites for the adsorption and degradation of Congo red, but also shortens the migration distance of photogenerated carriers, facilitating their separation and transfer at the heterojunction interface. The corresponding EDS elemental mapping (Fig. 2h) further confirmed that the Bi, V, O, Zn, S, and In elements were evenly distributed in the synthesized materials and highly dispersed in the selected area, indicating that strong interactions between BiVO_4_ and ZnIn_2_S_4_ were formed. This can accelerate the charge separation rate and mitigate the charge recombination rate during photocatalytic degradation.^31^

As shown in Fig. S1a, the XPS spectra of BiVO_4_/ZnIn_2_S_4_ confirmed that BiVO_4_/ZnIn_2_S_4_ was composed of Bi, V, O, Zn, In and S elements, consistent with the XRD results. All the XPS spectra were referenced to the C 1s peak (284.0 eV) to eliminate charge effects. In Fig. S1b, prominent peaks at 164.22 eV and 158.92 eV were assigned to Bi 4f_5/2_ and Bi 4f_7/2_,^32^ respectively. In Fig. S1c, the peaks at 1045.7 eV and 1022.5 eV are assigned to Zn 2p_1/2_ and Zn 2p_3/2_,^33^ respectively. In Fig. S1d, the peaks at 452.9 eV and 445.3 eV could correspond to In 3d_3/2_ and In 3d_5/2_,^34^ respectively. In Fig. S1e, weak peaks at 162 eV and 160 eV were attributed to the 2p_1/2_ and 2p_3/2_ of S,^35^ respectively. In Fig. S1f, the peaks at 524.35 eV and 516.46 eV can be assigned as the 2p_1/2_ and 2p_3/2_ of V, respectively, indicating the sample contained the pentavalent vanadium of BiVO_4_. Fig. S1g indicates that the O 1s XPS spectrum in BiVO_4_ could be divided into three characteristic peaks as the O–H (531.8 eV), V–O (530.5 eV), and Bi–O (529.3 eV) bonds. From Fig. S1a–g, the binding energies corresponding to Bi 4f, V 2p, and O 1s in the BiVO_4_/ZnIn_2_S_4_ composite shifted to higher energy levels compared with those of BiVO_4_, indicating the decrease in the electron density of BiVO_4_. Meanwhile, the binding energy of Zn 2p, In 3d, and S 2p in BiVO_4_/ZnIn_2_S_4_ shifts toward lower energy compared with those of ZnIn_2_S_4_, implying an increase in the electron density of ZnIn_2_S_4_. The binding energy shift provides essential evidence for the formation of the internal electric field (IEF)^36^ at the heterojunction interface: the difference in Fermi levels between BiVO_4_ and ZnIn_2_S_4_ causes charge rearrangement at the interface. Therefore, it can be concluded that the photogenerated electrons migrated from BiVO_4_ to the ZnIn_2_S_4_ in the BiVO_4_/ZnIn_2_S_4_ composite, which provides a strong driving force for the directional migration of photogenerated carriers, meanwhile, the photocatalytic performance was enhanced by accelerating the electron and hole separation.

BET analysis is a useful method to characterize the textural properties of photocatalysts.^37^ Table S1 shows that BiVO_4_/ZnIn_2_S_4_ exhibits the largest specific surface area and pore size. This creates additional active sites and facilitates charge transport, thus adsorpting more pollutants and accelerating the adsorption rate, which in turn improves the photocatalytic activity. Reduced charge transport distances and optimized surface characteristics are beneficial for heterogeneous photocatalytic processes.^38^

Fig. S2 presents the FT-IR spectra of pure BiVO_4_ and the BiVO_4_/ZnIn_2_S_4_ composite. For the pure BiVO_4_, the diffraction peak at 3440.36 cm^−1^ corresponds to the O–H stretching vibrations, which is possibly related to the adsorbed water or hydroxyl group.^39^ The diffraction peak of the V–O bond is located at 1382.14 cm^−1^, and the peak at 745.84 cm^−1^ is attributed to the bending vibration of the Bi–O and O=Bi–O bonds.^40^ For the BiVO_4_/ZnIn_2_S_4_ composite, the 3440.36 cm^−1^, 1403.69 cm^−1^, and 1087.65 cm^−1^ absorption peaks represent the vibration peaks of the O–H, Zn–S,^41^ and In–S bonds,^42^ respectively. Based on the analysis, the FT-IR spectra of the BiVO_4_/ZnIn_2_S_4_ composite exhibit the characteristic peaks of BiVO_4_ and ZnIn_2_S_4_; moreover, compared with the spectrum of pure BiVO_4_, the characteristic absorption peaks of the composite shifted toward higher wavenumber, suggesting that BiVO_4_ and ZnIn_2_S_4_ are not merely physically mixed.^43^

Through UV-visible diffuse reflection analysis of the catalysts, the optical absorption characteristics and *E*_g_ of the materials involved in the composite catalyst were determined according to the relationship between UV-visible light absorption coefficient and the *E*_g_ as follows: (*αhν*)^1/*n*^ = *K*(*hν* − *E*_g_),^44^ where *α* is the absorption coefficient, *h* denotes Planck's constant, *ν* represents the incident light frequency, *E*_g_ stands for the band gap, and *K* is a constant. The parameter *n* is determined by the transition properties of the semiconductor, where *n* = 1/2 and *n* = 2 correspond to direct and indirect transitions, respectively.^45^ Since BiVO_4_ is a direct transition semiconductor, *n* is 1/2.^46^ As shown in Fig. S5a, it can be seen that 520 nm and 560 nm are the optical absorption edges of BiVO_4_ and ZnIn_2_S_4_, respectively. However, the visible-light absorption range was significantly expanded after combining these two materials. The improvement stems from the broad visible-light absorption range of ZnIn_2_S_4_, which increases the absorption capacity of visible light, raises the visible-light utilization rate, and improves the photocatalytic performance. Fig. S5b shows that the *E*_g_ of BiVO_4_, ZnIn_2_S_4_, and BiVO_4_/ZnIn_2_S_4_ are 2.13 eV, 2.28 eV, and 1.93 eV, respectively. Since the photocatalytic activity largely depends on the *E*_g_ of the semiconductor photocatalyst, the BiVO_4_/ZnIn_2_S_4_ composite exhibits a higher catalytic activity. The point of zero charge (PZC) of pure BiVO_4_ is reported to be around pH 4.2,^47^ while that of pure ZnIn_2_S_4_ is around pH 6.0.^48^ The BiVO_4_/ZnIn_2_S_4_ composite was formed by combining the two semiconductors and thus will possess the both surface charge characteristics. Therefore, it can be inferred that the PZC of the BiVO_4_/ZnIn_2_S_4_ composite lies between these two values. The reaction pH in this study was 6.0; therefore, the surface of the composite is slightly negatively charged, which is not favorable for the adsorption of anionic Congo red. This indicates that the excellent photocatalytic performance of the composite is mainly attributed to efficient separation of photogenerated carriers and the generation of reactive radicals.

### 3.2 Photocatalytic activity

**Fig. 3:**
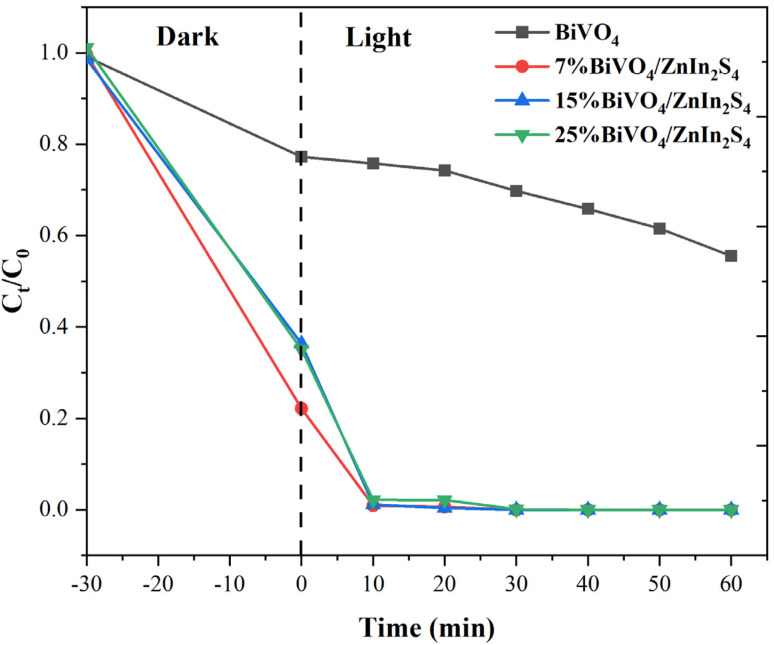
Degradation rate plot of Congo red by BiVO_4_ and BiVO_4_/ZnIn_2_S_4_.

Fig. 3 summarizes the decomposition of Congo red by pure BiVO_4_ and the BiVO_4_/ZnIn_2_S_4_ composites of different ratios. After 30 minutes of dark reaction, all BiVO_4_/ZnIn_2_S_4_ composites exhibited significantly higher degradation ability than that achieved with BiVO_4_. This was due to the constitutive Z-type heterojunction between BiVO_4_ and ZnIn_2_S_4_, which enhanced visible-light absorption and suppressed the recombination of photogenerated charge carriers, thereby improving the utilization efficiency of the photocatalyst. Thus, 7% BiVO_4_/ZnIn_2_S_4_ achieved 100% degradation efficiency after 10 minutes. However, increasing the BiVO_4_ loading led to a decline in the photocatalytic performance. The photocatalytic degradation of Congo red followed the pseudo-first-order kinetic model. The rate constant of pure BiVO_4_ was 0.0100 min^−1^, while those of 7% BiVO_4_/ZnIn_2_S_4_, 15% BiVO_4_/ZnIn_2_S_4_, and 25% BiVO_4_/ZnIn_2_S_4_ composites increased to 0.1514 min^−1^, 0.1022 min^−1^, and 0.1050 min^−1^, respectively, which were significantly higher than that of pure BiVO_4_. Furthermore, the stability of the composites was tested: after six consecutive usage cycles, the Congo red degradation rate was approximate 100% (Fig. S3), proving that the prepared composite samples had high stability. Although this study did not directly validate particle stability through XRD and XPS characterization, the six-cycle tests achieved systematic validation.^49^

### 3.3 Photocatalytic mechanism

**Fig. 4:**
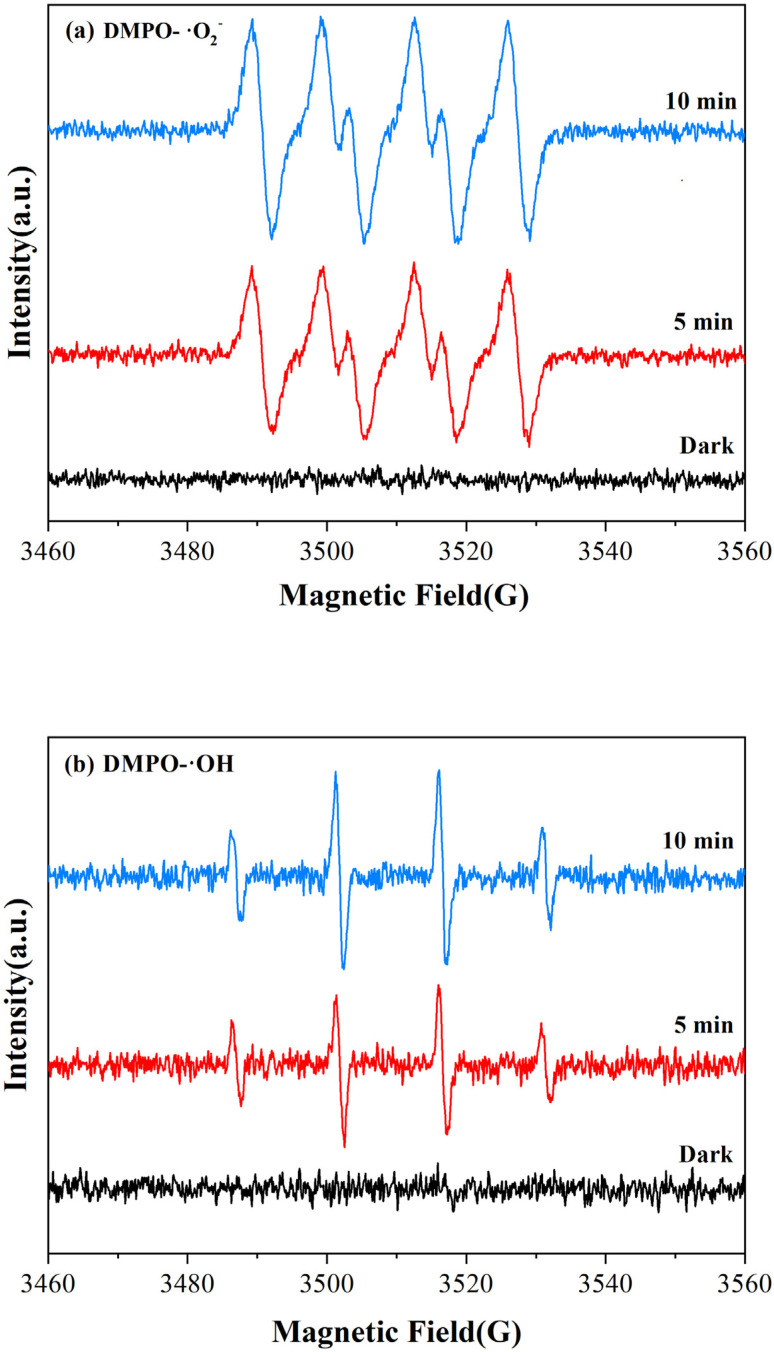
EPR plots of ˙O_2_^−^ (a) and ˙OH (b).

For the EPR measurements, 5,5-dimethyl-1-pyrroline-*N*-oxide (DMPO) was used as the trapping agent to detect ˙O_2_^−^ and ˙OH radicals. Fig. 4a and b demonstrate that no significant free radical signal was detected under dark conditions, while light irradiation induced the emergence of characteristic EPR signals corresponding to DMPO-˙O_2_^−^ and DMPO-˙OH adducts. This confirms that both ˙O_2_^−^ and ˙OH radicals were generated during the photocatalytic process.

Further confirming the main active species driving Congo red degradation during the photocatalytic process, adding TEOA barely affected the degradation efficiency (Fig. S4), suggesting that h^+^ was not the primary reactive species. In contrast, adding BQ and IPA suppressed the degradation rate, confirming that ˙O_2_^−^ and ˙OH radicals acted as the dominant reactive species under light irradiation, which is consistent with EPR results. The coexistence of ions in actual wastewater may affect degradation efficiency. According to previous studies, low concentrations of Na^+^, Ca^2+^, Mg^2+^, Cl^−^, and NO_3_^−^ show negligible impact on degradation, as evidenced by the lack of consumption of active free radicals.^50^ SO_4_^2−^ may exhibit slight inhibition only at concentrations exceeding 100 mmol L^−1^, which is significantly higher than typical wastewater levels. Furthermore, the Z-type heterojunction primarily utilizes ˙O_2_^−^ as the active species in this study, which is less susceptible to capture by CO_3_^2−^. This effectively circumvents the inhibition issues associated with conventional ˙OH systems;^51^ therefore, BiVO_4_/ZnIn_2_S_4_ shows promising application potential in complex ionic environments.

**Fig. 5:**
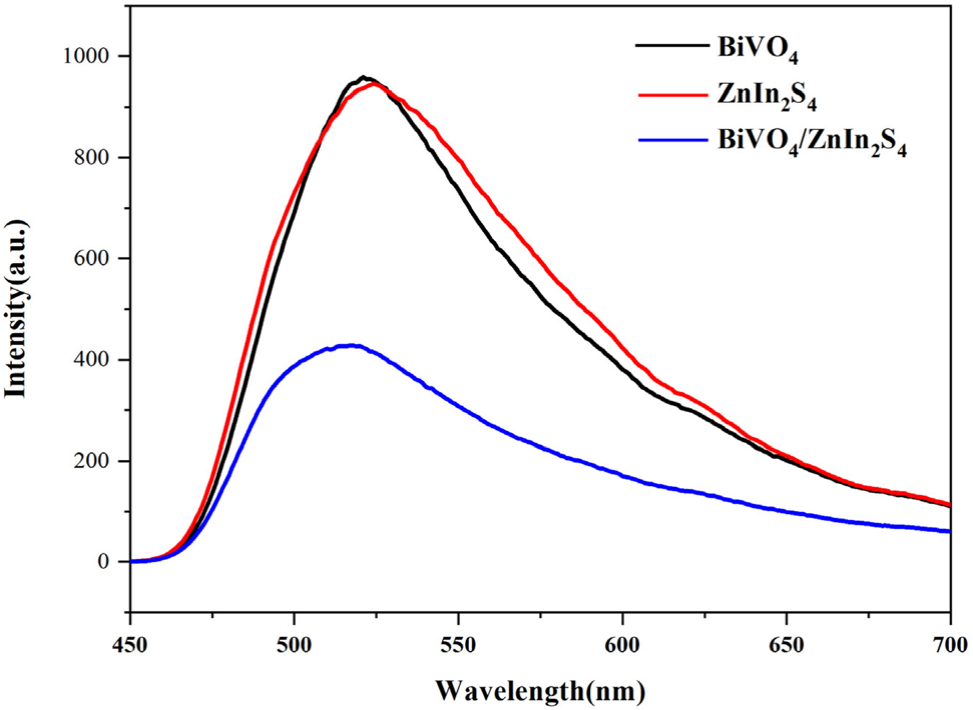
PL spectra of BiVO_4_, ZnIn_2_S_4_, and BiVO_4_/ZnIn_2_S_4_.

PL analysis is a powerful technique to evaluate the recombination efficiency of photogenerated electron–hole pairs, which plays a decisive role in determining the photocatalytic performance of materials.^52^ As presented in Fig. 5, the three materials showed similar linear PL spectra, indicating that ZnIn_2_S_4_/BiVO_4_ did not cause novel luminescence phenomena.^53^ The ZnIn_2_S_4_/BiVO_4_ composite exhibited a weak PL signal, indicating effective charge transfer took place at the interface of the composite. The interface effect suppressed the recombination of photogenerated charge carriers,^31^ which facilitated longer charge-carrier lifetime, enhanced the photogenerated carrier separation efficiency and improved the photocatalytic performance.

**Fig. 6:**
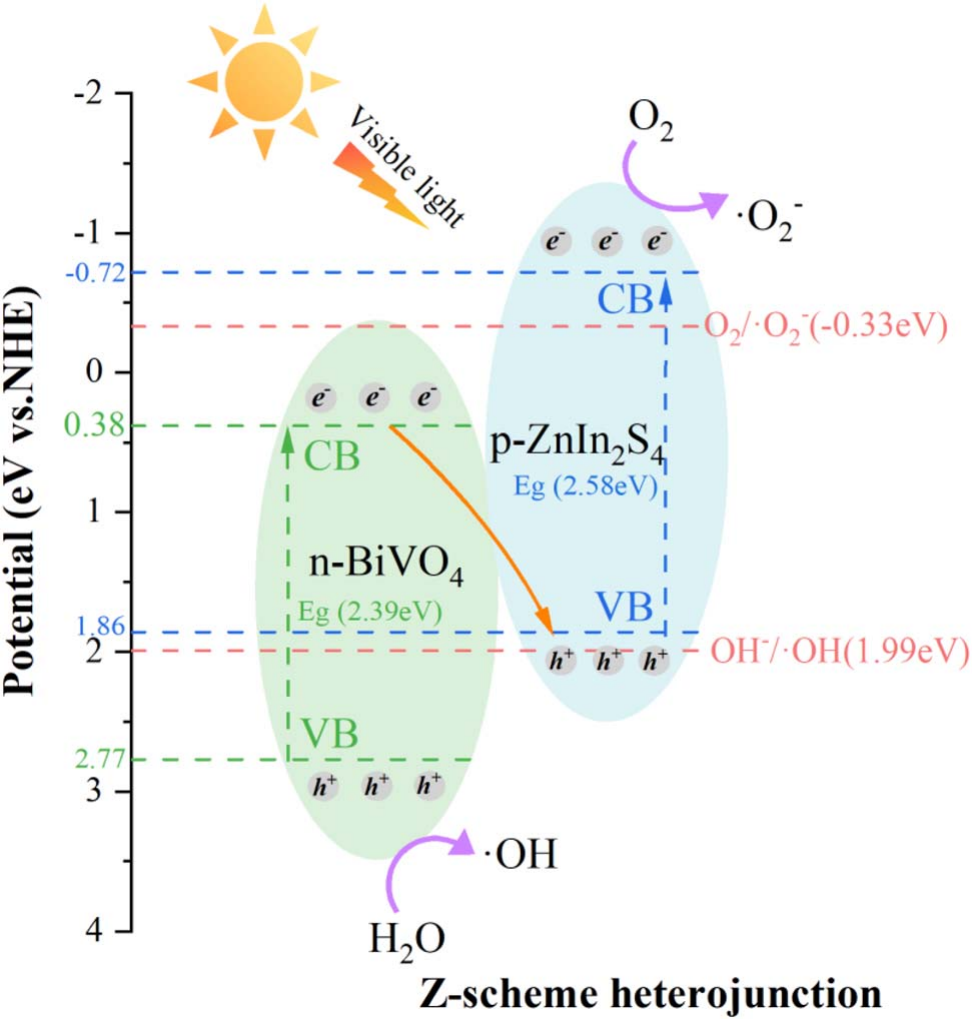
Degradation mechanism of pollutants by the BiVO_4_/ZnIn_2_S_4_ catalyst.

Notably, recent studies^23,24^ have proposed that the S-scheme heterojunction follows a more thermodynamically favorable electron-transfer pathway than the traditional Z-scheme. As shown in Fig. 6, the photocatalytic mechanism of BiVO_4_/ZnIn_2_S_4_ is consistent with the Z-scheme based on the above test results. Specifically, if the S-scheme pathway were followed, the strongly reducing electrons in the CB of ZnIn_2_S_4_ and strongly oxidizing holes in the VB of BiVO_4_ would recombine, leaving only weak redox carriers that cannot generate ˙O_2_^−^ and ˙OH radicals; however, these two radicals were detected in the radical trapping experiments. The VB of BiVO_4_ and ZnIn_2_S_4_ were 2.77 eV (ref. 34) and 1.86 eV, respectively, and the *E*_g_ of BiVO_4_ and ZnIn_2_S_4_ were 2.39 eV and 2.58 eV, respectively. Therefore, the CB of BiVO_4_ and ZnIn_2_S_4_ can be calculated from the equation *E*_CB_ = *E*_VB_ − *E*_g_,^54^ giving *E*_CB_ values of 0.38 eV and −0.72 eV, respectively. The VB of ZnIn_2_S_4_ was 1.86 eV, which is less positive than the 1.99 eV (potential of OH^−^/˙OH); in contrast, CB of BiVO_4_ (0.38 eV) is more positive than that of O_2_/˙O_2_^−^ (−0.33 eV).^55^ After electron–hole separation, e^−^ would react with O_2_ to generate ˙O_2_^−^, moreover, h^+^ was retained in the VB of BiVO_4_ and oxidized H_2_O to generate ˙OH.^56^

1 Photocatalyst + *hν* → e^−^ + h^+^

2 O_2_ + e^−^ → ˙O_2_^−^

3 OH^−^ + h^+^ → ˙OH

4 Congo red + (˙OH, ˙O_2_^−^) → degradation product

The toxicity of the end products of the photocatalytic degradation of Congo red depends on the degradation degree. It is generally accepted that the inorganic small-molecule products (CO_2_, H_2_O, inorganic ions, *etc.*) produced during the degradation of Congo red are basically non-toxic.^57^ In this experiment, the Z-scheme BiVO_4_/ZnIn_2_S_4_ photocatalyst achieved efficient deep mineralization of Congo red, so it can be reasonably inferred that the final degradation products are non-toxic.

## 4 Conclusion

Direct Z-scheme BiVO_4_/ZnIn_2_S_4_ composite photocatalysts were successfully prepared *via* a hydrothermal method and used to degrade Congo red. Among these composites, the 7%BiVO_4_/ZnIn_2_S_4_ photocatalyst exhibited the highest efficiency. The enhanced photocatalytic performance of the BiVO_4_/ZnIn_2_S_4_ composites was attributed to a direct Z-scheme charge-transfer mechanism, which effectively promoted charge separation and reduced electron–hole recombination. These findings highlight the potential of BiVO_4_/ZnIn_2_S_4_ composites as efficient and stable photocatalysts for environmental remediation. This work provides practical guidance for the design and fabrication of BiVO_4_-based Z-scheme heterojunction compositions in the photocatalytic field.

## Conflicts of interest

There are no conflicts to declare.

## Supplementary Material

RA-016-D6RA00765A-s001

## Data Availability

The data supporting this article have been included as part of the supplementary information (SI). Supplementary information: Table S1 and Fig. S1–S5. See DOI: https://doi.org/10.1039/d6ra00765a.
