# Supplementary material for: Enhanced photocatalytic degradation of Congo red by a BiVO4/ZnIn2S4 composite: performance and mechanism
Source: RSC Adv. 2026 Apr 27;16(24):21667–75. doi: 10.1039/d6ra00765a (PMC13112377; doi:10.1039/d6ra00765a)
Supplement: RA-016-D6RA00765A-s001 [file RA-016-D6RA00765A-s001.pdf]

Supplementary Information (SI) for RSC Advances.
This journal is © The Royal Society of Chemistry 2026

Supplementary information

Application of $BiVO_4/ZnIn_2S_4$ composite to photocatalytic degrade Congo red

Xinyue Liu[1,2], Yixin Zhang[2], Jiaxing Yu[2], Ming Li[2*]

[1]Nanjing-Helsinki Institute in Atmospheric and Earth System Sciences, Nanjing University, Suzhou 215163, China

[2]College of Forestry, Northeast Forestry University, Harbin 150040, China

[*]**Corresponding author:** Prof. Ming Li

College of Forestry, Northeast Forestry University, Harbin 150040, China

Tel: 86-451-82192120

E-mail: liming1986@nefu.edu.cn **(M. Li)**

Table S1 Specific surface area, pore size, and pore volume of $BiVO_4$, $ZnIn_2S_4$, and $BiVO_4/ZnIn_2S_4$

Catalyst	S_{BET} (m^2/g)	Pore size (nm)	Pore volume (cm^3/g)
$BiVO_4$	3.583	11.77	0.01055
$ZnIn_2S_4$	55.75	14.24	0.2199
$BiVO_4/ZnIn_2S_4$	61.75	15.28	0.2129

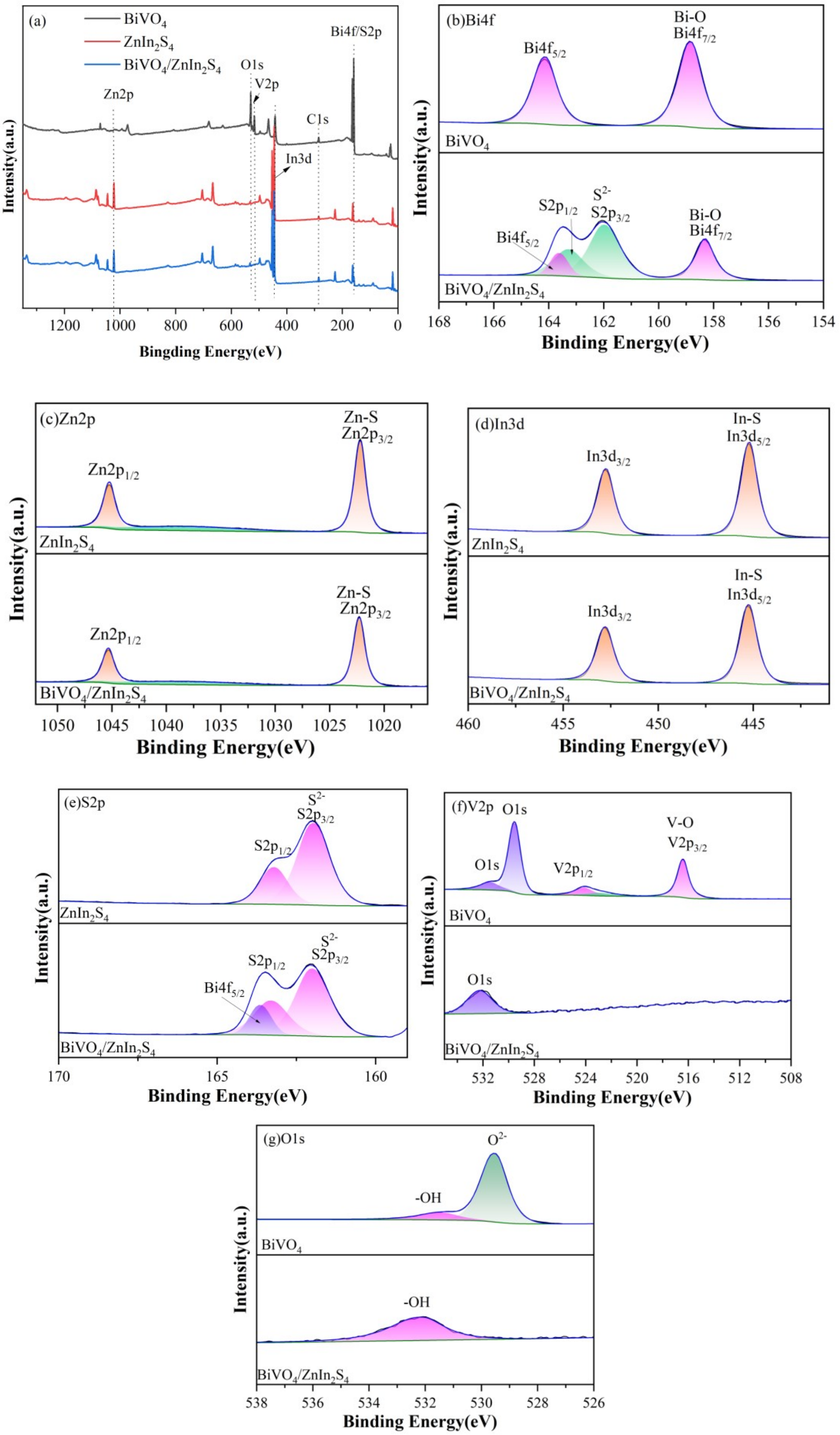

Fig. S1 XPS spectra of $BiVO_4$ and $BiVO_4/ZnIn_2S_4$: Full spectrum (a), Bi 4f (b), Zn 2p (c), In 3d (d), S 2p (e), V 2p (f), O 1s (g).

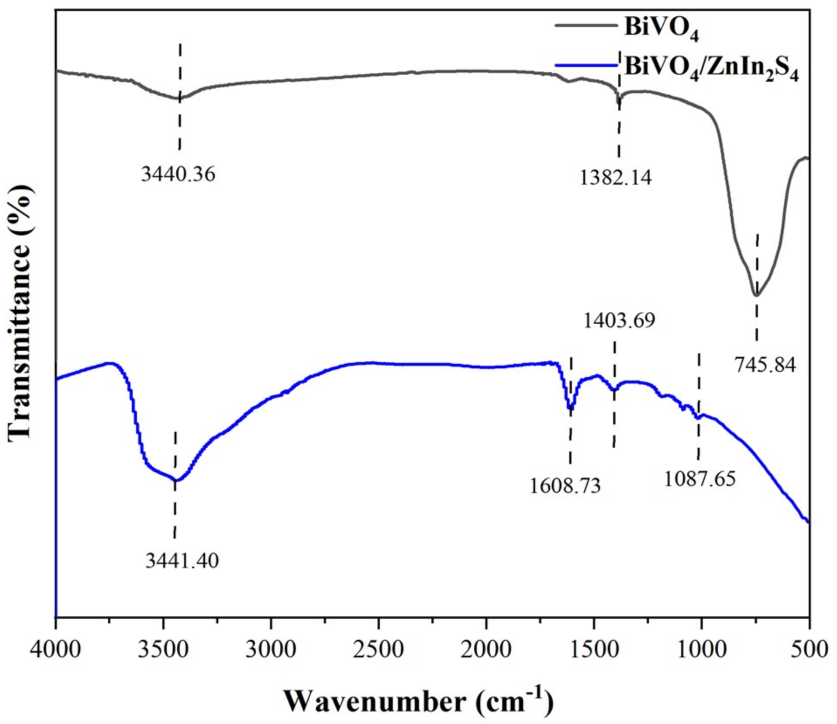

Fig. S2 FT-IR spectra of $BiVO_4$ and $BiVO_4/ZnIn_2S_4$

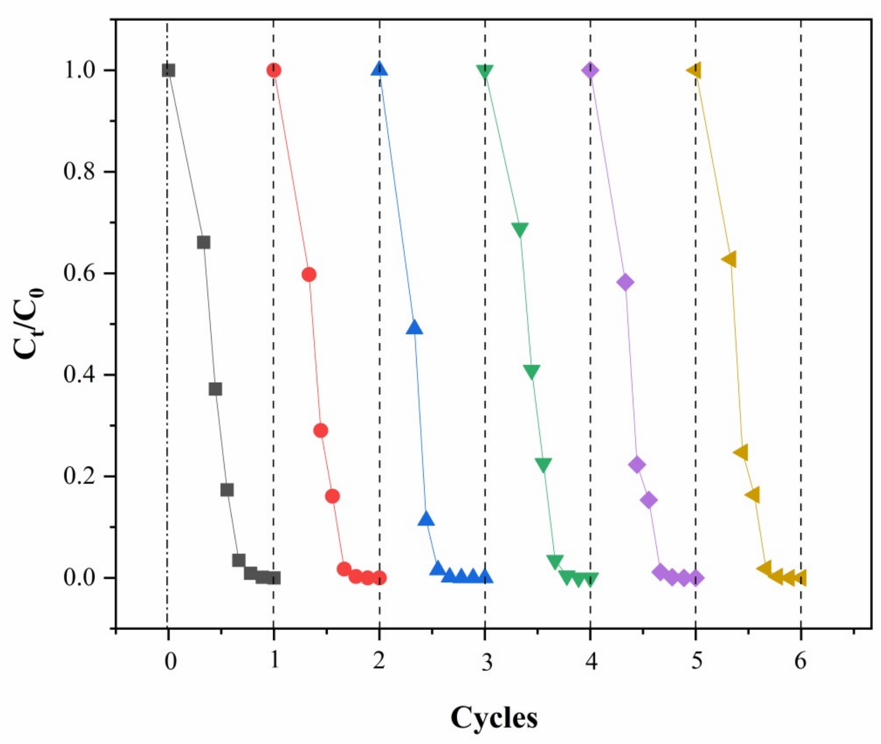

Fig. S3 Degradation efficiency of $BiVO_4/Znln_2S_4$ during six cycles

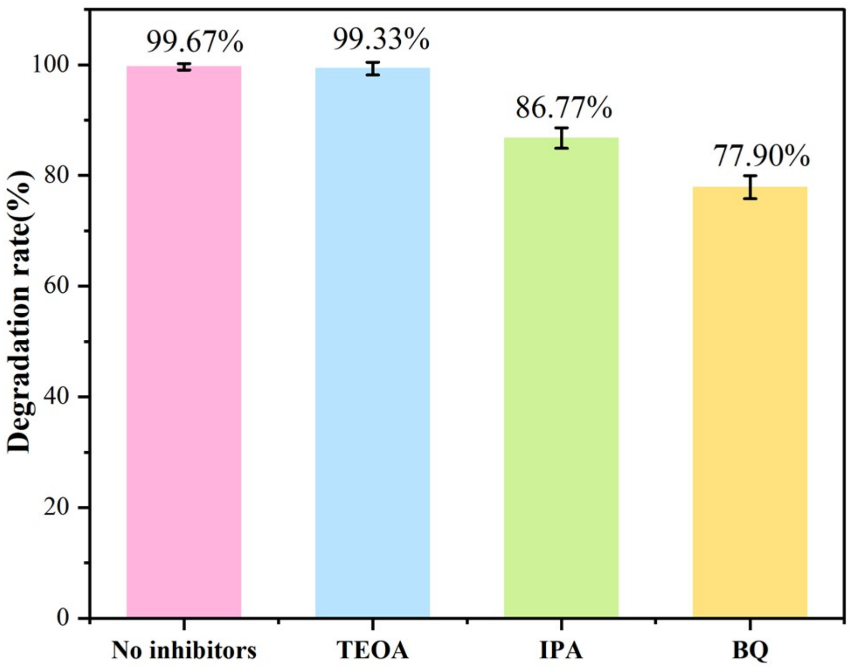

Fig. S4 Influence of scavengers on photocatalytic degradation rate of CR by 7% $BiVO_4/Znln_2S_4$

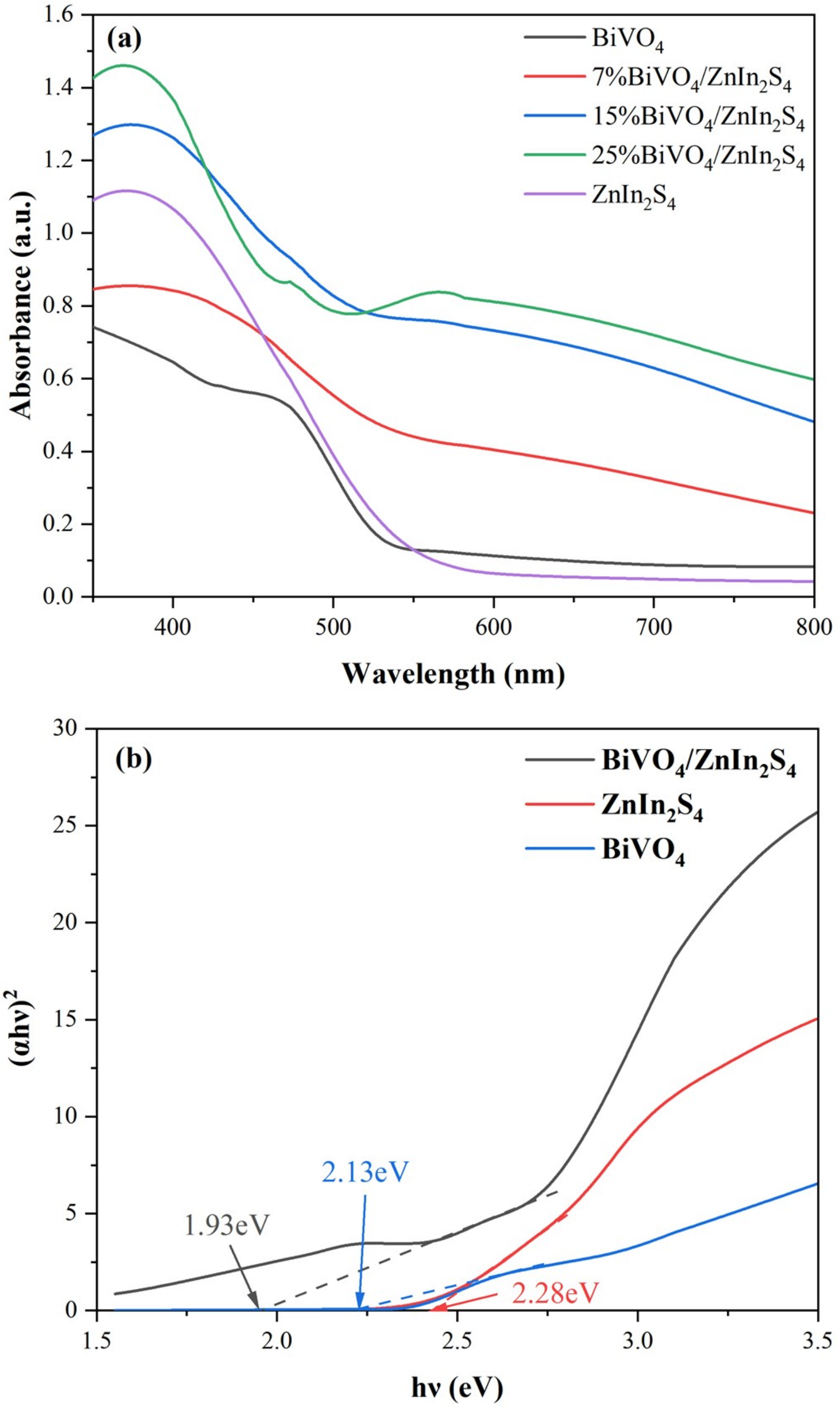

Fig. S5 Diffuse reflectance UV-vis spectra of samples (a), Plots of $(ah\nu)^2$ versus the photon energy ($h\nu$) of $ZnIn_2S_4$ and $BiVO_4$ with the corresponding Eg (b)
